# A New Developed Airlift Reactor Integrated Settling Process and Its Application for Simultaneous Nitrification and Denitrification Nitrogen Removal

**DOI:** 10.1155/2013/345725

**Published:** 2013-07-15

**Authors:** Tao Zhang, Chaohai Wei

**Affiliations:** ^1^The Key Lab of Pollution Control and Ecosystem Restoration in Industry Clusters, Ministry of Education, South China University of Technology, Guangzhou 510006, China; ^2^Key Laboratory of Environmental Protection and Eco-Remediation of Guangdong Regular Higher Education Institutions, College of Environmental Science and Engineering, South China University of Technology, Guangzhou 510006, China; ^3^College of Architectural and Surveying & Mapping Engineering, Jiangxi University of Science and Technology, Ganzhou 341000, China

## Abstract

This study presented the performance of simultaneous nitrification and denitrification (SND) process using a new developed hybrid airlift reactor which integrated the activated sludge reaction process in the airlift reactor and the sludge settling separation process in the clarifier. The proposed reactor was started up successfully after 76 days within which the COD and total nitrogen removal rate can reach over 90% and 76.3%, respectively. The effects of different COD/N and DO concentrations on the performance of reactor were investigated. It was found that the influent COD/N maintained at 10 was sufficient for SND and the optimum DO concentration for SND was in the range of 0.5 to 0.8 mg L^−1^. Batch test demonstrated that both macroscopic environment caused by the spatial DO concentration difference and microscopic environment caused by the stratification of activated sludge may be responsible for the SND process in the reactor. The hybrid airlift reactor can accomplish SND process in a single reactor and in situ automatic separation of sludge; therefore, it may serve as a promising reactor in COD and nitrogen removal fields.

## 1. Introduction

The environmental problems arising from nitrogenous compounds pollution, including oxygen depletion, toxicity to aquatic organism, and promotion of eutrophication, have attracted great attention in past decades [1]. Especially, some industrial wastewaters contain high concentration of ammonia nitrogen, such as coking wastewater, wastewater from fertilizer plant, and leachate. It is very important to remove nitrogen from these wastewaters before drainage. 

Biological nitrification and denitrification methods are the most widely used nitrogen removal methods [2, 3]. Nitrification requires an aerobic condition, whereas denitrification occurs under anoxic condition [4]. Thus, two-stage anoxic/oxic processes are generally used to meet the different condition requirements for nitrogen removal [1, 5]. However, many recent studies have demonstrated that these two steps for nitrogen removal can occur simultaneously in a single reactor, known as simultaneous nitrification and denitrification (SND) process [6–9]. Compared to conventional biological nitrogen removal process, SND can offer several advantages including simplifying the treatment system, reducing carbon source and alkalinity consumption, and saving aeration energy requirement [4]. 

The most widely used reactor for SND nitrogen removal is sequencing batch reactor (SBR) [10–12], because it enables the formation of the alternate aerobic and anoxic conditions in a time sequence manner. However, SBR is a kind of intermittent flow reactor which is not appropriate for continuous flow wastewater treatment. In recent years, airlift reactor (ALR), whose advantages include low energy requirement, effective mass transfer rate and mixing, elimination of dead volumes, and little footprint [13], has been used for the SND process in a continuous aeration and feed mode [2, 4, 14]. Moreover, the authors have also detected the phenomenon of total nitrogen removal in a field airlift reactor which is used for coking wastewater treatment Pan et al., [15].

Apart from the specific structure of the airlift reactor, keeping the sludge retention time (SRT) at relatively long time is also a prerequisite for efficient SND nitrogen removal because the growth rate of nitrifiers is very slow [1]. In order to prolong the SRT, either increasing the recycling ratio of sludge or reducing the unnecessary activated sludge loss from the effluent is feasible. However, increasing the recycling ratio will lead to low wastewater treatment rate and energy consumption, so reducing the sludge loss has been a considerable method. A membrane filter device has been introduced into the ALR by Meng et al. [14] for the purpose of withholding the activated sludge in the reactor, and nitrogen removal has been achieved. Nevertheless, the accumulation of recalcitrant compounds and soluble microbial products (SMPs) together with the membrane fouling has confined the application of membrane filter bioreactors [16].

Based on the above consideration, high efficiency settling process may be used as a replaceable method for membrane filter. So, a new reactor which integrates the ALR reactor and inclined plate settling reactor is developed, and it is named as hybrid airlift reactor (HALR) in this study. It is expected that the ALR can accommodate proper environmental condition for SND process while the new coupled clarifier can ensure the in situ separation of sludge from the effluent, so the whole reactor can maintain adequate SRT and accomplish the SND nitrogen removal independently. 

In this study, the proposed HALR reactor was used to investigate SND nitrogen removal ability. Furthermore, some factors which will influence the performance of the reactor were determined, such as controlled DO concentration and ratio of chemical oxygen demand to nitrogen (COD/N). Besides, the possible reasons for nitrogen removal in the proposed reactor were analyzed.

## 2. Materials and Methods

### 2.1. Experiment Setup

**Figure 1:**
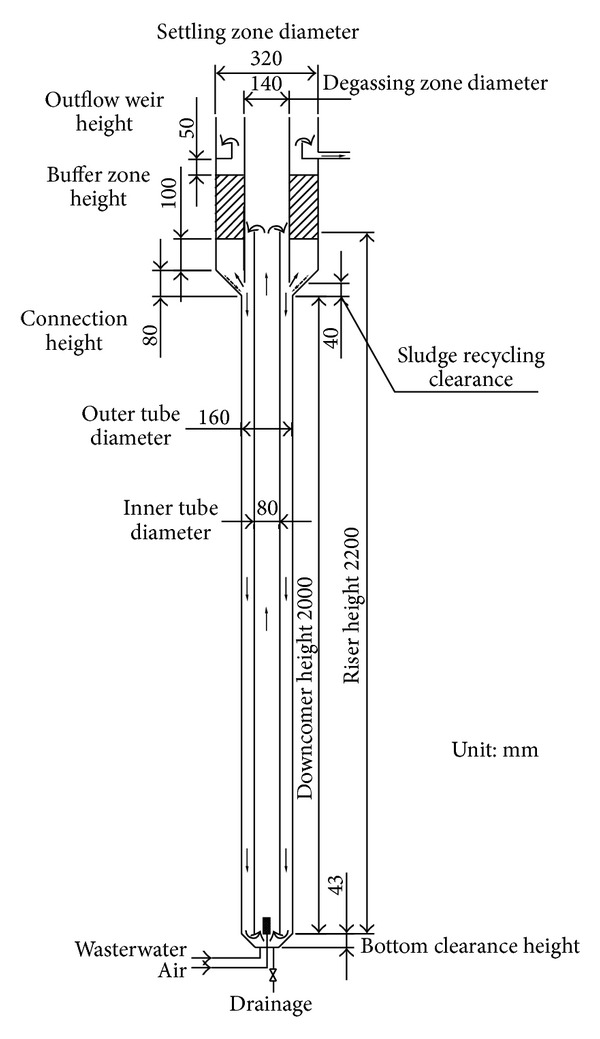
Schematic diagram of the experiment setup.

The schematic diagram of the experimental reactor is shown in Figure 1. The reactor was made of transparent Perspex with a working volume of 47.4 L. It can be divided into three zones: reaction zone, degassing zone, and settling zone. The reaction zone is composed of two concentric tubes with the inner diameter of 160 and 80 mm, respectively. There is a gas sprayer mounted at the bottom of the inner tube. When the reactor works, the gas bubbles from the sprayer move upward into the inner tube and drive the liquid circulation flow between the inner tube and the annule zone. The inner tube enables the liquid to move upward and is called the riser. The annule zone between the two tubes names as the downcomer in which the liquid moves downward. The heights of the riser and downcomer are 2200 and 2000 mm, respectively. The bottom conic height and angle are 43 mm and 45°, respectively. Enlarged degassing zone and settling zone are mounted at the top of the reaction zone and they are connected with the reaction zone through an 80 mm conic shape transition. Degassing zone is just at the top of the riser with a diameter of  140 mm. The settling zone is just at the outer side of the degassing zone, and it is packed with inclined plates. The length of these inclined plates is 230 mm, and they are arranged 60° from the horizontal direction and about 20 mm in perpendicular distance. There is a buffer zone with the height of 100 mm under the inclined plates. Outflow weir is 50 mm above the top of the inclined plate which can drain the treated wastewater. 

Although the riser is aerated, the downcomer is gas-free because nearly all bubbles are escaped from the free liquid surface of degassing zone if the superficial gas velocity is relatively low [2]. Thus, a spatial distribution of dissolved oxygen (DO) may be formed at the presence of oxygen utilization in the reactor, and this is beneficial particularly regarding its application for SND [14] because the SND process requires the formation of aerobic and anoxic environment concurrently in the same reactor. Integrating the inclined plate clarifier on the top of the reaction zone can form a compact reactor and accomplish the in situ separation of activated sludge. When the mixed liquid of reaction zone flows into the clarifying zone through a horizontal gap, it changes its flow direction and moves upward through the condensed sludge layer and inclined plates zone, then the sludge can be effectively withdrawn as a result of flocculation and settling. Separated sludge can automatically slide into the reaction zone and rejoin the biological reaction process. 

### 2.2. Operation Condition

Synthetic wastewater was used as influent in all experiments. It contained three primary macronutrients consisting of sodium acetate, ammonium chloride, and sodium bicarbonate and micronutrients consisting of 0.03 g L^−1^ KH_2_PO_4_, 0.045 g L^−1^ K_2_HPO_4 _, 0.15 g L^−1^ CaCl_2_, 0.3 g L^−1^ MgSO_4_·7H_2_O, 0.01 g L^−1^ FeSO_4_, and 0.0015 g L^−1^ MnCl_2_·4H_2_O. The concentrations of micronutrients were fixed throughout this study, but those of macronutrients were changed depending on the requirements of different experimental designs. The pH of synthetic wastewater was controlled in the range of  7.0 to 8.0 which was modulated by 1 M Na_2_CO_3_. 

The reactor was inoculated with activated sludge from local municipal wastewater treatment plant (Liede, Guangzhou, China) which was running in a modified anaerobic/anoxic/oxic process. The mixture sludge (20 L) taken from the anoxic tank (5.6 g MLSS L^−1^, 10 L) and oxic tank (5.2 g MLSS L^−1^, 10 L) was used as the seed inoculum. The reactor was continuously aerated with an air compressor whose flow rate can be adjusted and measured by a precalibrated air rotameter. The synthetic wastewater entered the bottom of the reactor by a peristaltic pump, and the flow rate was controlled and measured by a precalibrated liquid rotameter. DO and pH were monitored at the upper part of the reactor by a DO electrode (InPro6050, Mettler) and a pH electrode (InPro4010, Mettler), respectively. A transmitter (M300, Mettler) was used which allows the continuous recording of pH and DO data. Constant DO was maintained by frequently adjusting the valve of air rotameter. After activated sludge has accumulated to a certain concentration, excess sludge was withdrawn through the bottom valve periodically to maintain the sludge retention time (SRT) at about 25 days. The operation temperature was controlled at 28 ± 1°C. 

The run length of the HALR investigated has been lasted for 220 days in a continuous flow mode. In the first 76 days, the reactor was started up successfully and run steadily for a period of time. During the following 144 days, the effects of DO value and COD/N ratio on performance of SND were investigated. During the whole operating process, after each operation condition had been changed, at least three HRT cycles had been waited for reaching a relatively steady state. 

### 2.3. Analytical Methods

Concentrations of ammonium, nitrate, and nitrite in both influent and effluent were measured by spectrophotometry with commercial test kits (Hach, USA) after filtration of the samples through acetate filter device with pore size of 0.45 *μ*m. The COD, suspended solid (SS), and MLSS were analyzed according to the standard methods [17]. All samples were collected and analyzed at interval time of 48 h. The removal efficiency of total nitrogen (TN) can be calculated using the following expression:

(1) ηTN =[NH4+]in−([NH4+]out+[NO2−]out+[NO3−]out)[NH4+]in  ×100%,

where the subscript in and out represent the influent and effluent, respectively. 

## 3. Results and Discussion

### 3.1. Reactor Performance during the Start-Up and Steady-State Running Period

In order to accumulate the nitrifiers and to avoid the excessive growth of heterotrophic microorganism in the HALR reactor, HRT was controlled at about 24 h at the initial running stage, and the reactor was fed with the synthetic wastewater with a low C/N ratio (COD: 480 mg L^−1^, NH_4_^+^-N: 120 mg L^−1^). After being operated with an acclimation stage of 24 days, the reactor was amended with the synthetic wastewater with COD/N = 7 (COD: 840 mg L^−1^, NH_4_^+^-N: 120 mg L^−1^) and HRT was changed to 16 h. Then the reactor was run for another 52 days until it reached a steady state for a period of time. The DO concentration was controlled at 0.8 ± 0.05 mg L^−1^ throughout this period. 

**Figure 2:**
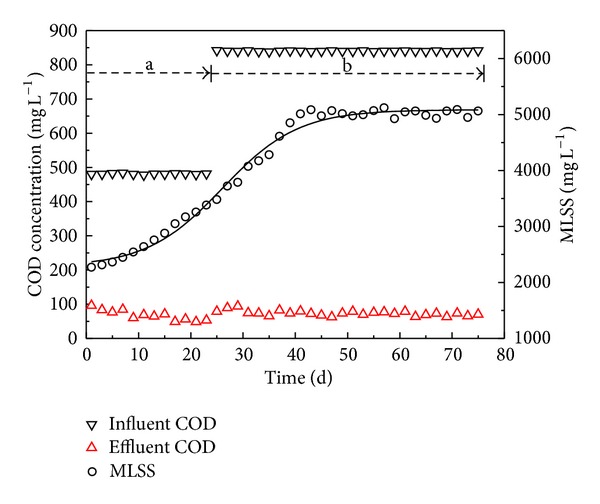
Concentration variation of COD and MLSS in startup and steady run period.

Figure 2 shows the variation of COD and MLSS concentrations as a function of the acclimation time. At the initial stage (a) with lower influent COD concentration, there was no obvious acclimation stage for COD removal and the effluent COD concentration was 67.5 mg L^−1^ on average. After 24-day operation, the COD loading was raised because higher COD/N wastewater was used in the stage (b). A dramatic increase in COD removal efficiency was observed, and approximately 90% of the initial COD was removed in this stage. The COD removal rate per unit volume of HALR is about 1.12 kg COD m^−3^ d^−1^ which is comparable to other reactors reported in the literature. Fu et al. [8] has reported that COD removal rate of 1.27 ± 0.21 kg COD m^−3^ d^−1^ has been achieved in a modified anoxic/oxic-membrane bioreactor (A/O-MBR) with a HRT of 1.5 d. Fdez-Polanco et al. [18] have obtained 80% COD removal efficiency and COD removal loading of 1.2 kg COD m^−3^ d^−1^ in a pilot scale anaerobic-aerobic fluidized bed reactor for the simultaneous carbon and nitrogen removal from municipal wastewater. 

In order to accumulate nitrifiers and raise the MLSS concentration, sludge was not discharged from the startup of the reactor until the MLSS concentration was beyond 5000 mg L^−1^. At the same time, sludge was efficiently separated from the effluent and slid downward automatically from the clarifier zone. At last, it was entrained and returned to the reaction zone, so that sludge concentration was always increasing before being discharged. From Figure 2, the MLSS concentration increased relatively slowly during the first 7 days and increased more quickly in subsequent days. In the 43th day, MLSS concentration reached a maximum value up to 5084 mg L^−1^. In the following days, a low concentration of active sludge was discharged through the bottom valve, and the typical concentration of biomass in the effluent is about 30 mg/L. The MLSS concentration in the reactor was maintained at about 5000 mg L^−1^, and the sludge retention time (SRT) of this system was at about 25 days. The typical sludge in the HALR reactor is conventional activated sludge flocs, and no obvious granular sludge has been observed. It may be because that relatively low aeration strength in the experiment leads to low shear stress in the reactor, which is not favorable for the formation of granular biomass. Besides, the settling ability of the sludge is good, and the sludge volume index (SVI) can be remained at about 90 mL/g. 

**Figure 3:**
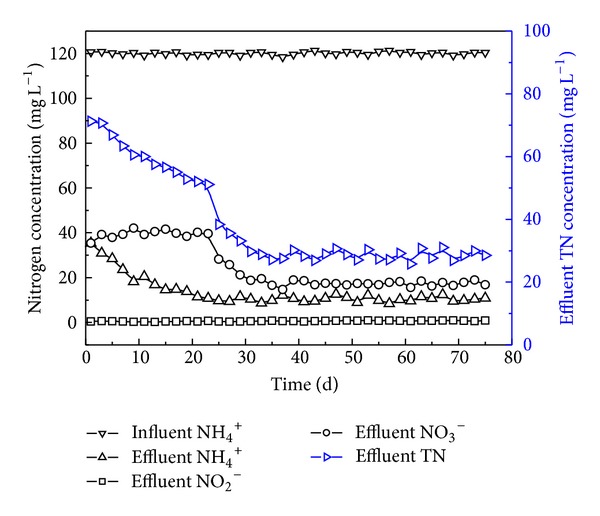
Profiles of different nitrogen concentrations in the startup and steady run periods.

Figure 3 shows the time-dependent variations of influent and effluent NH_4_^+^-N, NO_2_^−^-N and NO_3_^−^-N concentrations and effluent TN concentration. The influent NH_4_^+^-N concentration was always fixed at about 120 mg L^−1^ in this period. From the startup of reactor to day 24, the effluent NH_4_^+^-N concentration was gradually reduced with the time as it decreased from 35.4 to 10.8 mg L^−1^. Low nitrifying efficiency at the initial stage can be related to the low population of nitrifiers. The low COD/N wastewater used in stage (a) is favorable for the growth of autotrophic nitrifiers, because organics for the growth of heterotrophic microorganisms are limited. Therefore, nitrifiers can accumulate in the reactor, and nitrification effect can be raised gradually. From day 24 and onward, although the COD loading was increased, abundant nitrifiers were cultured, so that the efficiency of nitrification can be retained at a high level. The effluent NH_4_^+^-N concentration was 10.4 mg L^−1^ on average and the NH_4_^+^-N removal efficiency was over 90%. For all the 76-day operation, the nitrite concentration in the effluent was always below 1.0 mg L^−1^, indicating that no obvious nitrite accumulation occurred in the reactor. During the first 24 days, nitrate concentration in the effluent was 39.4 mg L^−1^ on average. However, from day 24 to 76, the nitrate concentration in effluent decreased rapidly with the increasing of influent COD concentration, and eventually the effluent nitrate reached a steady concentration of about 17.3 mg L^−1^.

**Figure 4:**
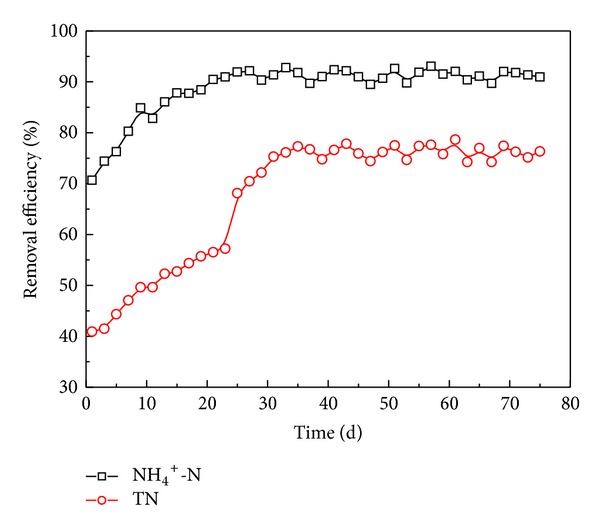
NH_4_^+^ and TN removal efficiency in the startup and steady run periods.

Figure 4 shows the calculated removal efficiency of NH_4_^+^-N and TN removal as a function of the acclimation time. It can be seen that the NH_4_^+^-N removal efficiency was increasing with the time at initial days and reached a steady level from day 24 onward. Nevertheless, the TN removal efficiency exhibited a different trend, and two obvious stages could be partitioned in association with the different influent COD concentrations. TN removal efficiency increased gradually at a low influent COD concentration but increased rapidly with the increasing influent COD concentration. Approximately 76.3% of TN was removed during the steady-state running period. It can be inferred that if carbon source for denitrification is sufficient, nitrification is the critical factor that limited the nitrogen removal efficiency as evidenced from the fact that both NH_4_^+^-N and TN removal efficiency increased gradually with the accumulation of nitrifiers in stage (a). However, the insufficient carbon source confined the denitrification process and resulted in lower TN removal efficiency and relatively higher nitrate concentration in the effluent. Once the COD/N was increased to 7, obvious increasing of TN removal efficiency was achieved because of the relative balance of nitrification and denitrification process. It should be noted that in the present condition the nitrogen removal contribution of microorganisms assimilation is about 7.6%, and it may lead to an overestimation for the effect of denitrification. The TN removal efficiency in this work was similar with the results reported in the literature. Li et al. [4] investigated the performance of different single-stage continuous aerated submerged membrane bioreactors (MBR) for nitrogen removal and achieved the removal of 94.2% ammonia nitrogen and 64.5% TN. Meng et al. [14] reported that 78% TN removal efficiency was obtained in an airlift internal circulation membrane bioreactor. When the TN removal rate per unit volume was considered, it could reach about 140 gN m^−3^ d^−1^ in HALR. Fu et al. [8] has achieved TN removal rate of 119.2 ± 22.1 gN m^−3^ d^−1^ in a modified anoxic/oxic-membrane bioreactor (A/O-MBR) with an HRT of 1.5 d. Farizoglu et al. [19] have acquired 99% TN removal efficiency at a removal loading rate of 17~436 gN m^−3^ day^−1^ in a jet loop membrane bioreactor. From these comparisons, it shows that the HALR with a relatively simple and compact structure can also achieve comparable TN removal rates to other reactors in the literature. 

### 3.2. Effect of COD/N on Reactor Performance

Denitrification is an anaerobic or anoxic biological process which is accomplished by heterotrophic microorganisms, and thus it is strongly dependent on the availability of organic carbon that serves as an electron donor of the process. For the proposed HALR, both aerobic carbon degradation microorganisms and anoxic denitrification microorganisms coexist in the reactor; accordingly, they compete for the limited available carbon source. This is the reason responsible for the relationship between the influent COD concentration and the effect of nitrogen removal. To disclose this relationship, four experiments with different COD/N ratios (COD/N = 4, 7, 10, 15) were carried out for an 80-day operation period. During these experiments, the DO was maintained at 0.8 ± 0.05 mg L^−1^, HRT was 16 h, MLSS was about 5000 mg L^−1^, and SRT was about 25 d. 

**Figure 5:**
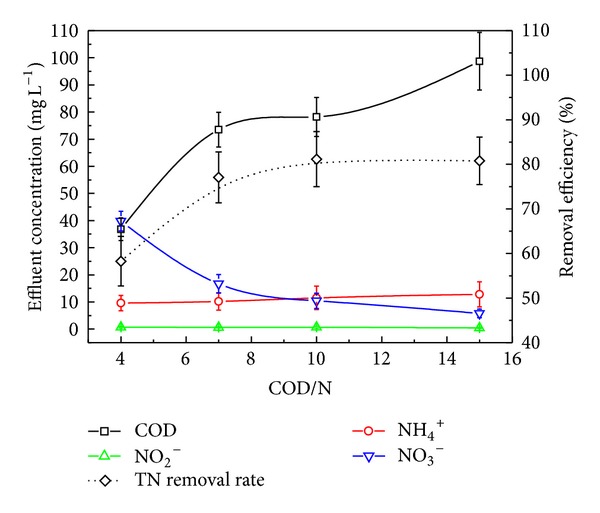
Reactor performance under different COD/N.

It can be seen from Figure 5 that the effluent COD concentration increased with the increasing of COD/N from 4 to 7. However, there was an insignificant change of the effluent COD concentration when the COD/N is changed from 7 to 10. The low value of effluent COD for COD/N = 4 is due to the lack of carbon source for denitrification, while the high value of effluent COD for COD/N = 15 is because that the influent was excessive. The NH_4_^+^-N and NO_2_^−^-N concentrations in the effluent were almost constant and were found to be about 10 mg L^−1^ and below 1 mg L^−1^, respectively. So, despite the variation of COD/N, the NH_4_^+^-N removal efficiency can remain at a stable level. The effluent NO_3_^−^-N concentration was reduced with the increase of COD/N but had a small change when COD/N was in the range of 7~15. The TN removal efficiency increased with the increasing of COD/N when COD/N is controlled below 10 but did not vary for further increase of COD/N. These results showed that when COD/N was over 10, the COD was sufficient for denitrification despite the competing of COD for aerobic microorganisms, and the TN removal efficiency was mainly determined by the nitrification effect of autotrophic microorganisms. The results of this experiment match well with the previous reports [14] in which COD/N = 10.04 was considered to be the optimal value for TN removal.

### 3.3. Effect of DO on Reactor Performance

DO is the critical factor which influences the occurrence possibility and specific rates of biological nitrification and denitrification processes. To determine the effect of DO on the performance of the SND process, the experiment with the varied DO concentration was performed for 64 days. Four different DO concentrations (0.3, 0.5, 0.8, and 1.2 mg L^−1^) were controlled sequentially in this period. The influent COD and NH_4_^+^-N concentrations were 840 and 120 mg L^−1^, respectively, which give COD/N as 7. The HRT was about 16 h, and the MLSS was controlled at 5000 mg L^−1^. 

**Figure 6:**
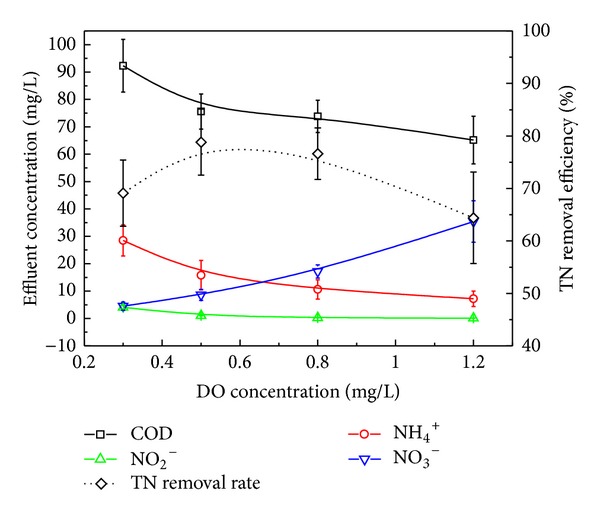
Reactor performance under different DO concentrations.

It can be seen from Figure 6 that the effluent COD concentration decreased with the increasing of DO concentration. The remaining COD was lowered to 92.3 mg L^−1^ and the COD removal efficiency can be over 89%. The increase in the DO concentration resulted in a decrease in the effluent NH_4_^+^-N concentration and an increase in the effluent NO_3_^−^-N concentration. The effluent NO_2_^−^-N concentration was almost independent of DO variations. Small accumulation of NO_2_^−^-N (<5 mg L^−1^) was detected when DO was below 0.3 mg L^−1^. For TN removal, the optimum DO control concentration was in the range of 0.5~0.8 mg L^−1^ and any deviations from this DO range reduced the TN removal efficiency. The optimum DO concentration was in accordance with the literature results. Pochana and Keller achieved up to 95% of the total nitrogen removal through SND under DO conditions between 0.3 and 0.8 mg L^−1^ in sequencing batch reactors [20]. Nakano et al. found that DO concentration enabling the highest SND performance was between 0.5 and 0.75 mg L^−1^ in a single reactor [21]. 

High DO concentration is favorable for nitrifiers but disadvantageous for anoxic denitrification process; therefore, in order to achieve the SND process in a single reactor, the DO concentration should be controlled in a properly middle level. For pure cultures of ammonium and nitrite oxidizers, the critical DO concentration below which nitrification does not occur is around 0.2 mg L^−1^; at the same time, denitrification can be ignored when the DO concentration is greater than 1.0 mg L^−1^ [22]. Taking into consideration DO concentration difference in riser and downcomer of HALR, it is reasonable to obtain optimum DO range between 0.5 and 0.8 mg L^−1^ for SND. 

### 3.4. SND Mechanism Analysis

In the available literature [4, 21, 23], two hypotheses are comprehensively accepted for explaining the mechanism of SND process: (1) macroscopic environment hypothesis that reveals the SND occurrence due to macroscale of different spatial DO concentrations in the reactor; (2) microscopic environment hypothesis that reveals the SND occurrence owing to the micro-scale via stratification of activated sludge or biofilm. To understand that both mechanisms may cocontribute to the removal of TN in the HALR, a batch experiment was conducted to demonstrate the effect of nitrogen removal when concentration gradient of DO in spatial distribution was excluded. 

**Figure 7:**
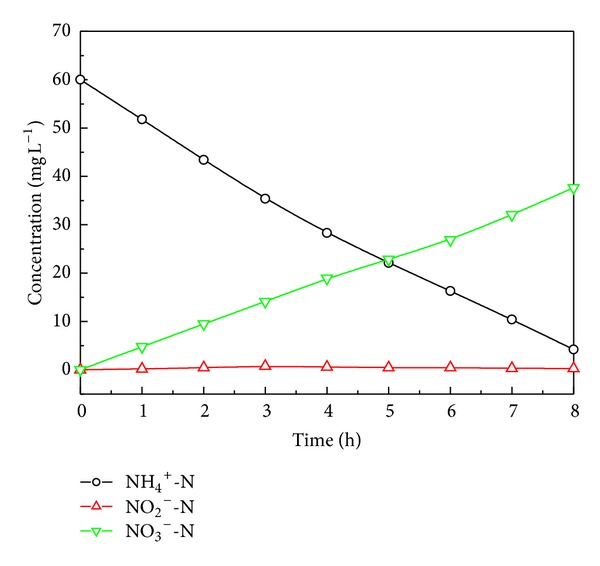
Variation of ammonium, nitrite, and nitrate in batch test.

In this batch test, three 1000 mL beakers were used as parallel reactors and they were all placed in water bath at 30°C. Each beaker was filled with 400 mL sludge which was taken out from the HALR and 400 mL synthetic wastewater with COD/N = 7. Thus, the initial concentration of COD and N in batch test are 420 and 60 mg L^−1^, respectively. A gas sprayer connected with air compressor was submerged at the bottom of beaker, and DO concentration was controlled at 0.8 ± 0.05 mg L^−1^. The initial MLSS concentration was 2476 ± 32 mg L^−1^, and pH was adjusted to 8.0. The batch test lasted for 8 h, and samples were taken out and analyzed immediately every hour for NH_4_^+^-N, NO_2_^−^-N, and NO_3_^−^-N. The variations of ammonium, nitrite, and nitrate are shown in Figure 7.

After 8 h operation, the NH_4_^+^-N concentration decreased from 60 to 4.2 mg L^−1^, the NO_2_^−^-N concentration was kept at a low value (<0.6 mg L^−1^) throughout the operating period, and the NO_3_^−^-N concentration increased from zero to 37.7 mg L^−1^. As the mass increase of the sludge between initial and after batch test was less than 2%, nitrogen removal by microorganism assimilation was neglected. It can be calculated that nearly 30% of the initial TN was removed by denitrification process. Because the beaker can be considered as a completely stirred tank reactor (CSTR), DO concentration in spatial distribution is homogeneous and the SND mechanism via macro-scale DO gradient is excluded. Therefore, for the batch test system, the possible reasons for SND can be explained by the fact that the micro-scale environment is effective for both nitrification and denitrification. According to the results ahead, the total SND nitrogen removal efficiency is 76.3% in HALR when COD/N is 7, subtracting the SND contribution of micro-scale, then the SND contribution of macro-scale is over 46.3%. To sum up, the SND mechanism in HALR includes both macroscopic and microscopic environment hypotheses.

## 4. Conclusion

An HALR reactor which integrated biological reaction in conventional internal loop airlift reactor and sludge separation in inclined plate clarifier was developed for the purpose of simultaneous carbon and nitrogen removal. Operated with synthetic wastewater, the HALR was successfully started up and reached a steady status after 76 days, during which both COD and NH_4_^+^-N removal efficiency were over 90% and TN removal efficiency was 76.3% on average. The TN removal efficiency increases when COD/N is increased from 4 to 10. However, it exhibits an insignificant variation with further increase in COD/N. DO was demonstrated as another critical factor influencing the SND nitrogen removal performance. DO concentration in the range of 0.5 to 0.8 mg L^−1^was preferable for nitrogen removal. Batch test demonstrates that when DO concentration gradient in spatial distribution was excluded, only about 30% TN removal efficiency can be achieved, which indicates that both macroscopic and microscopic environment mechanisms govern the SND process in the proposed HALR. The HALR can accomplish SND process in a single reactor and in situ automatic separation of sludge. At the same time, it is simple in structure, energy saving, and high efficient in COD and nitrogen removal. Therefore, it may serve as a promising reactor in the field of wastewater treatment.

## References

[1] He S-B, Xue G, Wang B-Z (2009). Factors affecting simultaneous nitrification and de-nitrification (SND) and its kinetics model in membrane bioreactor. *Journal of Hazardous Materials*.

[2] Dhamole PB, Nair RR, D’Souza SF, Lele SS (2009). Simultaneous removal of carbon and nitrate in an airlift bioreactor. *Bioresource Technology*.

[3] Jin R-C, Zheng P, Mahmood Q, Zhang L (2008). Hydrodynamic characteristics of airlift nitrifying reactor using carrier-induced granular sludge. *Journal of Hazardous Materials*.

[4] Li YZ, He YL, Ohandja DG, Ji J, Li JF, Zhou T (2008). Simultaneous nitrification-denitrification achieved by an innovative internal-loop airlift MBR: comparative study. *Bioresource Technology*.

[5] Guo H, Zhou J, Su J, Zhang Z (2005). Integration of nitrification and denitrification in airlift bioreactor. *Biochemical Engineering Journal*.

[6] Helmer C, Kunst S (1998). Simultaneous nitrification/denitrification in an aerobic biofilm system. *Water Science and Technology*.

[7] Chan YJ, Chong MF, Law CL, Hassell DG (2009). A review on anaerobic-aerobic treatment of industrial and municipal wastewater. *Chemical Engineering Journal*.

[8] Fu Z, Yang F, An Y, Xue Y (2009). Simultaneous nitrification and denitrification coupled with phosphorus removal in an modified anoxic/oxic-membrane bioreactor (A/O-MBR). *Biochemical Engineering Journal*.

[9] Münch EV, Lant P, Keller J (1996). Simultaneous nitrification and denitrification in bench-scale sequencing batch reactors. *Water Research*.

[10] Blackburne R, Yuan Z, Keller J (2008). Demonstration of nitrogen removal via nitrite in a sequencing batch reactor treating domestic wastewater. *Water Research*.

[11] Chiu Y-C, Lee L-L, Chang C-N, Chao AC (2007). Control of carbon and ammonium ratio for simultaneous nitrification and denitrification in a sequencing batch bioreactor. *International Biodeterioration and Biodegradation*.

[12] Zhan X, Healy MG, Li J (2009). Nitrogen removal from slaughterhouse wastewater in a sequencing batch reactor under controlled low DO conditions. *Bioprocess and Biosystems Engineering*.

[13] Jin R-C, Zheng P, Mahmood Q, Zhang L (2008). Performance of a nitrifying airlift reactor using granular sludge. *Separation and Purification Technology*.

[14] Meng Q, Yang F, Liu L, Men F (2008). Effects of COD/N ratio and DO concentration on simultaneous nitrifcation and denitrifcation in an airlift internal circulation membrane bioreactor. *Journal of Environmental Sciences*.

[15] Pan X, Li Y, Huang H, Ren Y, Wei C (2009). Biodegradation of thiocyanate and inhibitory interaction with phenol, ammonia in coking wastewater. *Journal of Chemical Engineering*.

[16] Xia S, Guo J, Wang R (2008). Performance of a pilot-scale submerged membrane bioreactor (MBR) in treating bathing wastewater. *Bioresource Technology*.

[17] APHA (1998). *Standard Methods For the Examination of Water and wasteWater*.

[18] Fdez-Polanco F, Real FJ, Garcia PA (1994). Behaviour of an anaerobic/aerobic pilot scale fluidized bed for the simultaneous removal of carbon and nitrogen. *Water Science and Technology*.

[19] Farizoglu B, Keskinler B, Yildiz E, Nuhoglu A (2007). Simultaneous removal of C, N, P from cheese whey by jet loop membrane bioreactor (JLMBR). *Journal of Hazardous Materials*.

[20] Pochana K, Keller J (1999). Study of factors affecting simultaneous nitrification and denitrification (SND). *Water Science and Technology*.

[21] Nakano K, Iwasawa H, Ito O, Lee T-J, Matsumura M (2004). Improved simultaneous nitrification and denitrification in a single reactor by using two different immobilization carriers with specific oxygen transfer characteristics. *Bioprocess and Biosystems Engineering*.

[22] Lin Y, Tay J, Liu Y, Hung Y (2009). Biological nitrification and denitrification processes. *Biological Treatment Processes*.

[23] Meng L, Bando Y, Nakamura M (2004). Development of rectangular airlift bubble column installed with support material for enhancement of nitrogen removal. *Journal of Bioscience and Bioengineering*.

